# Instagram as a Tool to Improve Human Histology Learning in Medical Education: Descriptive Study

**DOI:** 10.2196/55861

**Published:** 2025-02-19

**Authors:** Alejandro Escamilla-Sanchez, Juan Antonio López-Villodres, Carmen Alba-Tercedor, María Victoria Ortega-Jiménez, Francisca Rius-Díaz, Raquel Sanchez-Varo, Diego Bermúdez

**Affiliations:** 1 Department of Human Physiology, Human Histology, Anatomical Pathology and Physical and Sports Education Faculty of Medicine University of Malaga Malaga Spain; 2 IBIMA Bionand Platform Biomedical Research Institute University of Malaga Malaga Spain; 3 Unit of Anatomical Pathology University Hospital Virgen de la Victoria Malaga Spain; 4 Department of Public Health and Psychiatry Faculty of Medicine University of Malaga Malaga Spain; 5 Centre for Networked Biomedical Research in Neurodegenerative Diseases Madrid Spain

**Keywords:** medical education, medical students, histology, pathology, e-learning, computer-based, social media, Instagram, Meta, community-oriented, usability, utility, accessibility

## Abstract

**Background:**

Student development is currently taking place in an environment governed by new technologies and social media. Some platforms, such as Instagram or X (previously known as “Twitter”), have been incorporated as additional tools for teaching and learning processes in higher education, especially in the framework of image-based applied disciplines, including radiology and pathology. Nevertheless, the role of social media in the teaching of core subjects such as histology has hardly been studied, and there are very few reports on this issue.

**Objective:**

The aim of this work was to investigate the impact of implementing social media on the ability to learn human histology. For this purpose, a set of voluntary e-learning activities was shared on Instagram as a complement to traditional face-to-face teaching.

**Methods:**

The proposal included questionnaires based on multiple-choice questions, descriptions of histological images, and schematic diagrams about the subject content. These activities were posted on an Instagram account only accessible by second-year medical students from the University of Malaga. In addition, students could share their own images taken during the laboratory practice and interact with their peers.

**Results:**

Of the students enrolled in Human Histology 2, 85.6% (143/167) agreed to participate in the platform. Most of the students valued the initiative positively and considered it an adequate instrument to improve their final marks. Specifically, 68.5% (98/143) of the student body regarded the multiple-choice questions and image-based questions as the most useful activities. Interestingly, there were statistically significant differences between the marks on the final exam (without considering other evaluation activities) for students who participated in the activity compared with those who did not or barely participated in the activity (*P*<.001). There were no significant differences by degree of participation between the more active groups.

**Conclusions:**

These results provide evidence that incorporating social media may be considered a useful, easy, and accessible tool to improve the learning of human histology in the context of medical degrees.

## Introduction

Social media platforms are web-based technologies particularly suited to facilitate the exchange of ideas through collaboration, interaction, and discussion. The accessibility and low cost of internet access, together with the high number of users of these platforms, make social media one of the easiest and most effective ways to disseminate information. In fact, 4.65 billion people, equivalent to 58.4% of the world’s population, use social media [1]. In addition, most current medical students are far more knowledgeable and experienced with emerging technologies than preceding generations. Unlike traditional media (journals or television), social media emphasizes interactivity, motivation through social connections, and immediacy [2]. In this sense, the “social constructivism theory” states that interaction and socialization may help students learn and construct their knowledge and personal learning processes, supporting the use of social media for educational activities as a different tool for teaching and learning [3]. For all these reasons, social media platforms have progressively been incorporated into health care and medical education [4].

Technological advances have enabled rapid dissemination of medical updates through social networks such as Facebook, X (previously known as Twitter), or Instagram. Thus, students often have access to significant amounts of information, including content taught during traditional classes. Nevertheless, this information has not always been rigorously verified or is outdated, representing a formative disadvantage for medical students. Moreover, there is a lack of engagement and even dropout from classes because traditional education methodology is considered by the student body to be boring, unnecessary, or repetitive. Therefore, the faculty must adapt to meet their specific needs, changing traditional teaching styles and implementing new e-learning technologies [5].

Recently, the COVID-19 pandemic forced teaching staff to move further into a virtual education environment and highlighted the importance of communication between educators and learners through social media platforms. The rapid and efficient dissemination of information during the pandemic illustrated the significant influence of social media in the dissemination of medical literature and knowledge, not only among health care professionals but also among the student body [6,7]. For instance, Chan et al [8] demonstrated the benefits of using tools such as infographics posted on social media platforms (such as X and WeChat) to educate frontline health care workers about respiratory tract management and infection control in the setting of COVID-19. Thus, the pandemic prompted a paradigm shift in learning for students and medical residents by using different platforms (eg, YouTube, Zoom, Microsoft Teams) as e-learning tools under the new circumstances. Although face-to-face teaching is possible and desirable today, the use of social networks as educational instruments must continue with apps such as Instagram and aim to share image-based educational content to complement the classes.

Histology has long been an integral part of the medical curriculum [9] and continues to provide key information about biological tissues, physiology, and disease; it is therefore highly valued in clinical medicine and research. Furthermore, histopathology is a fundamental tool for diagnosis and prognosis. In addition, a thorough knowledge of histology is necessary for the surgical field and in general practice. However, histology and its nomenclature can be complex to understand for novice medical students, and consequently, it is often perceived as a secondary subject without clinical relevance [10]. From a pedagogical point of view, one of the main goals of histology courses is to ensure that students acquire the competencies necessary to understand histophysiology. For example, histology requires students to develop pattern recognition skills. Specifically, they must be able to identify what they are observing based on specific histologic features. Consequently, histology courses commonly include laboratory practices for students to train and develop these abilities. In this context, the study of histology through digital imaging might be a relevant alternative for the development of their curricula.

Instagram is a social networking service owned by Meta Platforms Inc that was launched in October 2010. Instagram allows photo and video sharing accompanied by text. The information can be shared either publicly or privately. Followers can archive shared posts, and the account’s owner can track the number of people reached and give feedback to their followers. The literature shows that this social network is being used for educational purposes in medical schools, predominantly in imaging-related subjects such as radiology [11], ophthalmology [12], dermatology [13], anatomy [14], fertility [15], pathology [16], plastic surgery [17], dentistry [18], and (with very few proposals) in histology [19]. Understanding how students interact with these novel social media–based teaching environments and their approaches during e-learning processes is a matter of high relevance [20]. On the other hand, there is a lack of evidence on how the use of social networks impacts the learning and follow-up of Spanish medical students in the first years of their formation in the field of histology. In the first courses, the curricula of a Spanish medical degree include a basic thematic area with fundamental core subjects to obtain the essential knowledge for the subsequent study of pathological alterations. Among these subjects, some necessarily require the use of images, such as anatomy, cytology, histology, and microbiology. For that purpose, an educational experience was carried out using the social network Instagram to make the subject more attractive to the students of the official degree of Medicine at the University of Malaga in Spain during the 2022-2023 academic year. Our main objective was to test whether the use of Instagram might facilitate knowledge acquisition and increase engagement with histology, leading to a positive impact on students’ qualifications. Additionally, we aimed to elucidate which type of visual material was more useful for medical students. Finally, we determined students’ perceptions of the integration of this tool in medical education.

## Methods

### Content of Histology in the Degree of Medicine at the University of Malaga

According to the syllabus for the degree in Medicine at the University of Malaga, histology is divided into 2 subjects (Human Histology 1 and 2) that are taught during the first and second years, respectively. The different didactic content is distributed sequentially, progressively increasing the theoretical difficulty (Table 1). First-year students learn general histology along with some special histology topics (eg, the immune system). The remaining systems and organs are studied during the second school year in the subject Human Histology 2. Some of the potential skills to be developed during these courses are knowledge about the architecture, morphology, and function of the different tissues or systems; recognizing the morphology and structure of tissues by microscopy and imaging techniques; and how to handle basic laboratory equipment and methodology. In addition, our curricula include the acquisition of some transversal competencies such as the capacity for analysis and synthesis, problem-solving or critical reasoning, and analysis, together with other abilities and skills (autonomous work, information management, and oral or written communication skills).

**Table 1:** Curricula content of the human histology subjects in the degree in Medicine at Malaga University.

Content			Issues dedicated to each topic, n
**Human Histology 1**			
	Tissues (epithelial, muscle, osseous, connective, nervous)	17	
	Stem cells	1	
	Blood and hematopoiesis	3	
	Circulatory system	1	
	Immunity and lymphoid tissues	5	
**Human Histology 2**			
	Digestive system	6	
	Respiratory system	2	
	Urinary system	2	
	Genital apparatus	6	
	Tegumentary system	1	
	Endocrine system	6	
	Nervous system and neurosensorial organs	12	

### Sample Size

This study was carried out with 167 students enrolled in the subject Human Histology 2 in the degree in Medicine at the University of Malaga during the 2022-2023 academic year. The final examination was performed by most of the students (153 students), of which 143 participated until the end of the Instagram experience. Thus, only 10 (6.5%) of the 153 involved students did not follow our account.

### Design of the Instagram Profile

After downloading the free app on a smartphone, a private Instagram account (username: @histologiauma) was created for the subject Human Histology 2 at the University of Malaga. The Instagram profile was linked to an institutional email address created to receive questions and comments from the student body of this subject. Students were notified of the availability of this account and were informed about the procedure to participate. For instance, they had to register by giving their real first name and last name. Once we checked they belong to the subject, the students were accepted as followers of @histologiauma.

### Virtual Microscope Images

The images published in @histologiauma belong to the image bank of the Histology Unit of the Department of Human Physiology, Human Histology, Anatomical Pathology and Physical-Sports Education of the Medical School at the University of Malaga. During the COVID-19 pandemic, we introduced a highly interactive, web-based digital microscope system to view histological images during online practical lessons, either from classroom or personal computers. This virtual microscope is currently based on the digitalization of 66 slides, providing the element of real-time dynamic microscopy and offering students a truly innovative experience at exceptionally high resolution. Interestingly, this virtual microscope offers the possibility to capture specific tissue areas and use these pictures to formulate specific questions.

### Account Content Feed

Two software applications were used to design the images: Canva and Microsoft Office PowerPoint. The free basic mode of Canva offers access to thousands of templates and 1 million free photos. Both applications allow drag-and-drop operations familiar to both average users and design professionals and feature templates, photo filters, images, icons, and shapes useful for customizing histological images (eg, including different shapes to highlight structures or cells within a tissue, adding numbers or letters).

**Figure 1:**
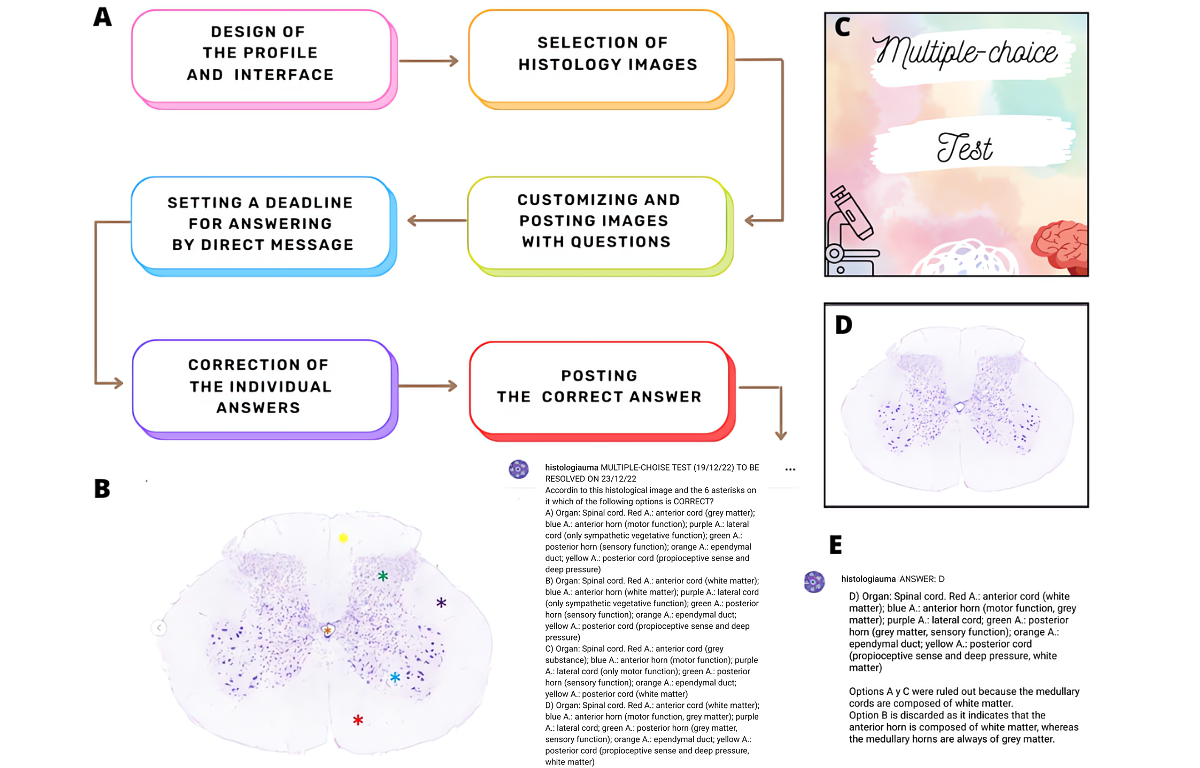
(A) Workflow for uploading a post in @histologiauma account, (B) screenshot of a post from @histologiauma account, (C) multiple-choice test interface, (D) image of a spinal cord transversal section with Klüver-Barrera staining, and (E) a student’s direct message including a reasoned correct answer.

During a 3-month trial period, 35 posts were published, of which 5 were announcements about the account rules. The general process for uploading new content to @histologiauma account is summarized in Figure 1.

### Ethical Considerations

The repository of digital images is composed of scanned slides with anonymized tissue remnants from Virgen de la Victoria University Hospital, whose patients provided signed informed consent for educational purposes.

The account @histologiauma was created as a private profile to be exclusively accessed by those second-year students of the degree in Medicine at the University of Malaga who voluntarily requested to participate. Images displaying captures from @histologiauma have been edited to make students’ profiles unidentifiable. Moreover, all the surveys were anonymously filled out by students.

The manuscript is a retrospective case report that does not require ethics committee approval at our institution since no demographic nor clinical data from patients were used.

### Type of Questions Posted on @histologiauma

The following sections were included in the Instagram platform for human histology education.

#### Image-Based Multiple-Choice Questions

**Figure 2:**
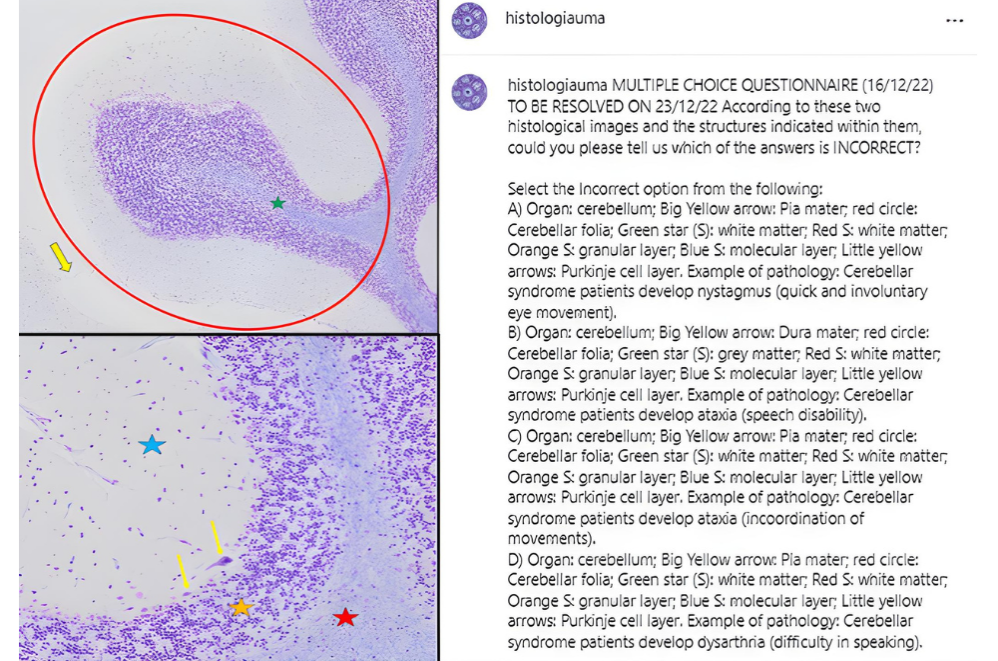
Screenshot of an image-based multiple-choice question about the cerebellum from the account @histologiauma.

There were 13 posts with image-based multiple-choice questions (Figure 2). Histological images of several organs studied during the academic year were posted. Different structures or cells were highlighted with arrows or other shapes (eg, stars, circles, asterisks). Multiple-choice questions with a single correct answer were posted, and 4 options were marked with labels A, B, C, and D in each question. For example, Figure 2 shows a post that asked students to select the incorrect option from the following answer choices: “A) Organ: cerebellum; Large yellow arrow: Pia mater; Red circle: Cerebellar folia; Green star: white matter; Red star: white matter; Orange star: granular layer; Blue star: molecular layer; Small yellow arrows: Purkinje cell layer. Example of pathology: Cerebellar syndrome, nystagmus as a quick and involuntary eye movement is included. B) Organ: cerebellum; Large yellow arrow: Dura mater; red circle: Cerebellar folia; Green star: gray matter; Red star: white matter; Orange star: granular layer; Blue star: molecular layer; Small yellow arrows: Purkinje cell layer. Example of pathology: Cerebellar syndrome, ataxia as a problem to speak is included. C) Organ: cerebellum; Large yellow arrow: Pia mater; red circle: Cerebellar folia; Green star: white matter; Red star: white matter; Orange star: granular layer; Blue Star: molecular layer; Small yellow arrows: Purkinje cell layer. Example of pathology: Cerebellar syndrome, ataxia or incoordination of movements is included. 4) Organ: cerebellum; Large yellow arrow: Pia mater; red circle: Cerebellar folia; Green star: white matter; Red star: white matter; Orange Star: granular layer; Blue star: molecular layer; Small yellow arrows: Purkinje cell layer. Example of pathology: Cerebellar syndrome patients develop dysarthria (difficulty in speaking).” The correct answer is B.

No negative scores were given in the case of wrong answers. All questions had a single correct answer. Each student provided their answer, including a brief justification in the form of a private message on Instagram. They were then notified about their success or encouraged to try again in case of failure. The answers were made public 5 days later in a comment, accompanied by a summary of the most common mistakes.

#### Descriptions or Questions Associated With Histological Images

**Figure 3:**
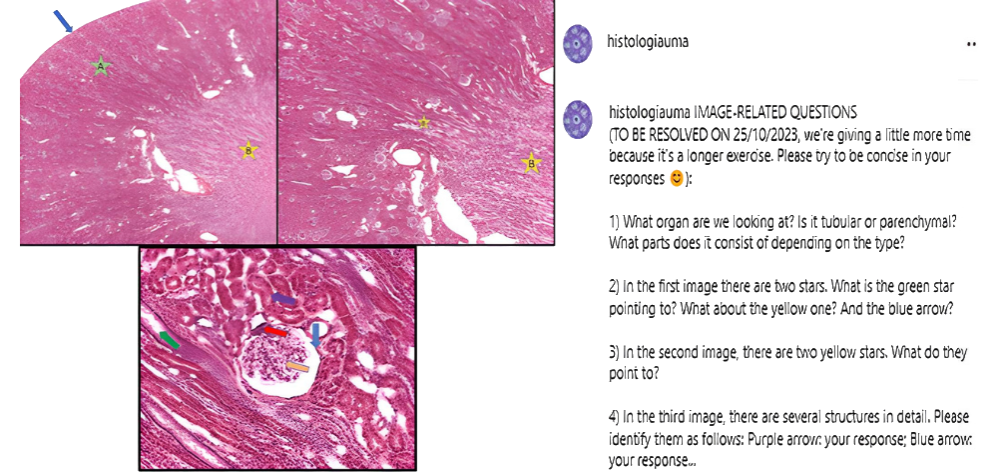
Screenshot of a histological image section (kidney) with the accompanying questions.

This section, consisting of 10 posts, showed histological images pointing out different structures and components to be identified by the students. For example, Figure 3 shows a post that included the following questions: “1) Identify the organ shown in the image. Is it a tubular or a parenchymatous organ? 2) What is the green star (A) pointing at? And the yellow star (B)? And the blue arrow?” Occasionally, comparisons between pathological and healthy tissues were posted, along with an introduction to clinical medicine. This section was conceived in accordance with the curricular competency entitled “From Histology to Medicine,” which aims to highlight the clinical aspects of human histology. The correct answer was published 5 days later as a comment on the post, and feedback was given to the students, as explained for the multiple-choice questions.

#### Didactic Schemes

**Figure 4:**
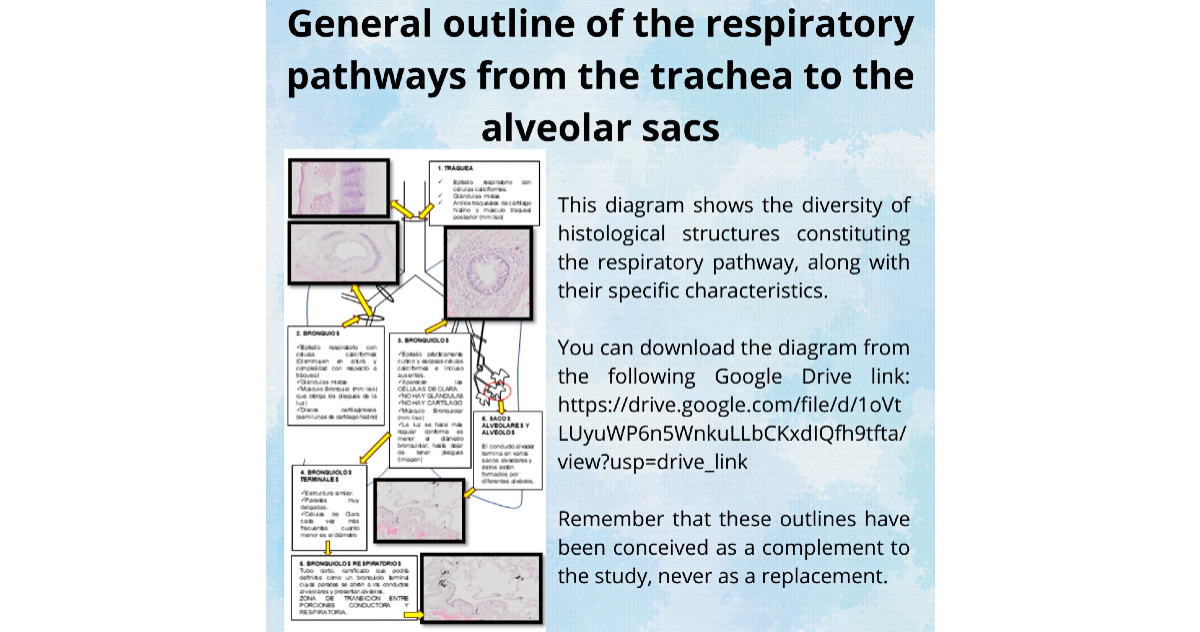
Screenshot of a scheme requested by students about the respiratory tract.

This section included 7 posts based on student requests for visual or explanatory diagrams of the content they found most difficult. Teachers then prepared specific outlines based on these requests, avoiding the inclusion of new content. An example is shown in Figure 4. Diagrams were created using free-design and educational software, such as PowerPoint or Canva and stored in a shared Google Drive folder. The link to access the content was posted on the Instagram account and made available for 1 week. The content of these diagrams was derived from the theoretical material already provided to the students, as they were conceived as a complementary tool to the study. Students were also encouraged to make their own schemes to learn how to summarize concepts.

#### “Do It Yourself” Section

**Figure 5:**
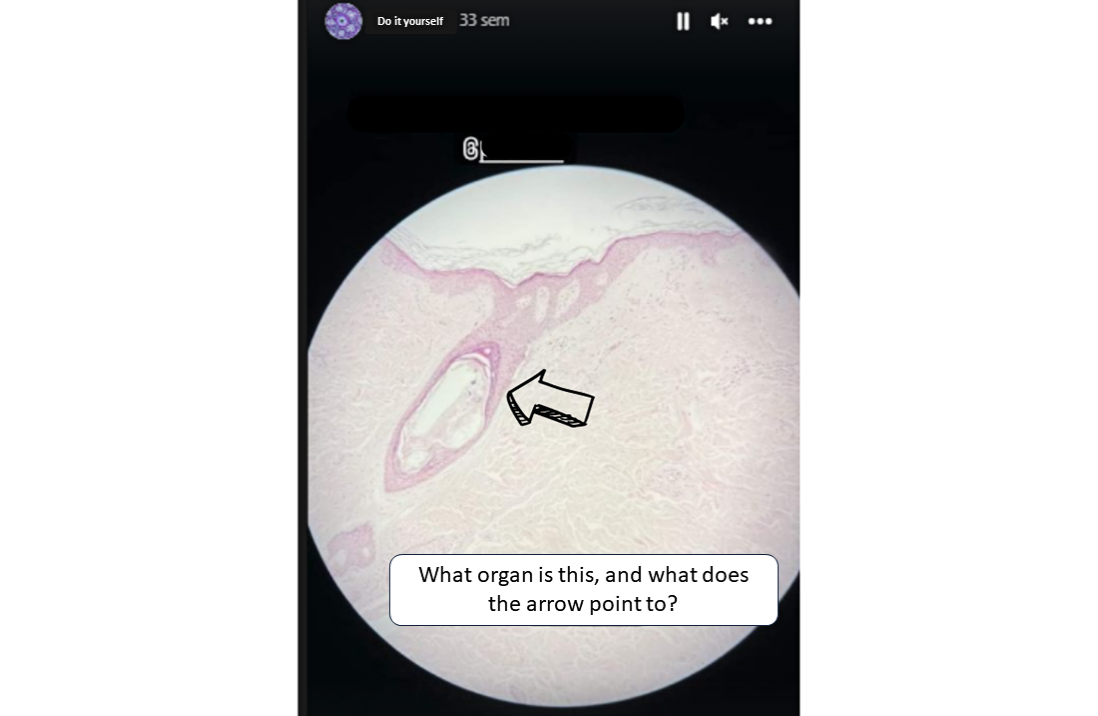
Screenshot of a “Do it yourself” section depicting a histological section of skin that was taken and elaborated on by one of the students.

During the practical classes, students were encouraged to take images of histological slides through the eyepiece of the microscopes with their own smartphones. Later, they posted the images for 24 hours in the form of a story on @histologiauma accompanied by a specific question to be solved by their classmates. In total, 25 images were shared as stories, and an example is shown in Figure 5.

#### Teaching Commitment

The teaching staff played a crucial role in providing feedback to the students, notifying them about their successes or mistakes through private messages and designing diagrams. Overall, 1 hour of work was required daily during the experience.

### Rating

The activity was conceived as a voluntary pilot study. Students who actively participated could add a maximum of up to 0.50 points to their final mark, regardless of whether their interventions were successful. Thus, students earned points in proportion to their level of participation. A score of 10 was assigned to students who answered 100% of the questions. The student body was organized in 4 groups according to the rewards received on @histologiauma (group 1=0-4.4 points; group 2=4.5-6.5 points; group 3=6.5-8.5 points; group 4=8.5-10 points). Therefore, these points served as an indicator of participation.

### Evaluation of the Activity as an Educational Innovation

To explore the influence of this innovative learning tool, data on student engagement and perceptions were collected during the lectures and at the end of the course through a final evaluation. The results were gathered throughout the academic semester with 3 anonymous surveys using a 5-point Likert scale. Each topic in the surveys covered a gradient of agreement with the statement presented (1=strongly disagree, 5=strongly agree). All questions were designed in Spanish by members of the teaching staff (AES, RSV, and DB) and later translated into English for publication.

At the beginning of this educational experience, the opinions of students about the inclusion of new technologies and the implementation of social media in our medical school were assessed through a first survey (called the pre-experience survey; Textbox 1).

This first survey also included specific questions regarding students’ perceptions of the use of Instagram in the histology course (Textbox 2).

The second survey (middle experience; Textbox 3) was conducted 2 months after the start of the project. This questionnaire focused on the general operation of the account and their early perceptions of the experience. The third and final survey contained the same questions (Textbox 3) and was carried out during the last week of theory classes.

**General questions in the initial survey (pre-experience).**

1. The university's educational systems are up to date and adapted to the times.
2. Most teachers use social networks as an educational resource.
3. Currently, the use of alternative educational tools is essential.
4. Bringing the basic subjects of a medical degree closer to the reality of professional practice is essential.
5. In general, teachers are concerned about updating educational tools.

**Specific questions for the initial survey (pre-experience).**

1. Instagram facilitates access to educational content on histology.
2. Accessing Instagram allows me to consult the content anywhere at any time (eg, bus, train).
3. Test questions are a useful tool to review theoretical or practical content.
4. Downloading the subject outlines helps me to complete histology concepts.
5. I expect to improve my academic grades thanks to this academic experience.

**Specific questions for the 2nd (mid-experience) and 3rd (end of the experience) surveys.**

1. I followed the @histologiauma updates daily.
2. I answered test questions.
3. I answered questions shared in the @histologiauma stories.
4. I shared some photographs in the “Do it yourself” section.
5. I answered the image-associated questions.
6. I used @histologiauma when traveling by public transport.
7. I used @histologiauma during class exchanges.
8. The test questions were adequate.
9. The schemes were useful.
10. We received highly personalized attention.
11. Circle the most useful sections of @histologiauma: “Do it yourself,” image-associated questions,” “multiple-choice questions,” “schemes.”
12. I will use @histologiuma to prepare for my final exams.

**Statistical Analysis**

Raw data from the survey responses were collected and analyzed using SPSS v.24 (IBM Corp). Descriptive statistics were used to characterize the data, with a frequency study carried out for each of the variables evaluated in the different surveys. Homoscedasticity (equality of variances) and normal distribution of the data were checked. Data corresponding to students’ marks (score range 0-10) are expressed as mean (SD). Mann-Whitney tests were conducted to compare overall grades from different cohorts (groups from different academic years or current cohort with the extra points versus the cohort without the extra points). The comparisons between the marks of the different groups (previously categorized into groups 1-4 according to the level of participation) and the degree of acquired knowledge evidenced by the final exam (global marks) were conducted using 1-way ANOVA followed by a post hoc Bonferroni test. Pearson correlation coefficients were calculated using the individual scores according to the students’ participation in @histologiauma and their final grades. An *r*>1 indicated a positive linear correlation between the 2 variables. The significance was set at 95% of confidence.

## Results

### Participant Demographics

From the very first post, 73 of 167 students enrolled in Human Histology 2 followed the account. At the end of the learning experience, there were 143 followers (143/167, 85.6%), of which 106 students had actively participated during the entire period. Analytics from these 143 students showed that 76.2% (109/143) were women and 23.8% (34/143) were men. Most (133/143, 93%) of them were 18 years to 22 years old and in their second year of the medical degree (139/143, 97.2%). There were only 4 repeaters of this subject, who were concomitantly in the third year of the degree. Full-time students represented 97.9% (140/143) of the respondents. The full demographic profile of the students is shown in Table 2.

**Table 2:** Student demographic data (N=143).

Characteristics		Results, n (%)
**Gender**		
	Female	109 (76.2)
	Male	34 (23.8)
**Age (years)**		
	18-22	133 (93)
	23-26	7 (4.9)
	27-30	2 (1.4)
	31-40	1 (0.7)
**Academic year**		
	2	139 (97.2)
	3	4 (2.8)
**Enrollment**		
	Complete	140 (97.9)
	Partial	3 (2.1)

### Pre-Experience Test About Students’ Perceptions of the Use of Social Media and New Technologies in Medical School Curricula

Of the participants, 83.9% (120/143) considered that the current educational system requires a significant update. Thus, 97.9% (139/143) of them strongly believed that the use of social networks should be significantly improved. Of the students, 99% (141/143) considered that using alternative educational tools is relevant, and 77.1% (110/143) of the students agreed that the use of social media such as Instagram facilitates access to the didactic content. Thereby, 95% (133/143) of the respondents found the subject more accessible thanks to @histologiauma, and 99.5% (142/143) believed they might improve their academic marks thanks to this experience.

### Survey About the Students’ Perceptions During the Experience

Multiple-choice questions (65/143, 45.7%) and image-based questions (33/143, 22.8%) were the students’ favorite sections, with 96.5% (138/143) and 99.3% (142/143) of the students, respectively, considering them highly useful for learning the subject. In contrast, the schemes and “Do it yourself” sections were the favorite sections for 12.6% (18/143) and 0.8% (1/143), respectively, of the students. The remaining 18.1% (26/143) reported a stated preference for combinations of the different sections (multiple-choice questions + image-based questions: 17/143, 11.8%; multiple-choice questions + schemes: 5/143, 3.9%; image-based questions + schemes: 3/143, 2.4%). No other activities were included in the experience. Additionally, 96.7% (138/143) of the students felt well-supported and guided by the teaching staff throughout the experience. In addition, students showed no preference between public transport or the exchange of classes for visualizing the didactic content available on Instagram.

### Impact of the Experience on Final Marks

The average grade obtained by the students from the Histology course during the academic year prior to the implementation of the experience (2021-2022: n=158 students) was 6.49 (SD 1.87) out of 10, whereas marks from the 2022-2023 cohort were significantly higher (mean 7.13, SD 1.68; *P*<.002; n=153), regardless of the extra points for participating in the experience. Once the earned points were included, the final outcome was not significantly different (2022/2023 without extra points: mean 7.29, SD 1.70; 2022/2023 with extra points: mean 7.13, SD 1.68; *P*=.03). Furthermore, the mean final grade from the 4 previous academic courses showed homogeneity in terms of having lower results (mean 6.12, SD 0.27; n=628 students) in comparison to our cohort. Overall, our data support that the use of social media produced a positive impact on students’ performance, even without considering the points for participating in @histologiauma.

Interestingly, a positive linear correlation between individual participation scores and final marks (not including the extra reward points) was found (*r*=0.439, *P*<.001). Moreover, the ANOVA showed significant differences between students’ marks according to their degree of participation (*P*<.001; Table 3). There was a trend of higher ratings according to the level of participation. The Bonferroni test showed that group 1 (the least engaged group with 0-4.4 points) achieved significantly lower global mean scores than the other 3 groups (all *P*<.001)*.* Finally, there were no significant differences among groups 2 to 4 (all *P*=.99).

**Table 3:** Students’ global marks (without the extra points) and rating obtained according to the degree of participation in @histologiauma.

Group	Global score, mean (SD)
Group 1 (0-4.4 points)	5.43 (2.65)
Group 2 (4.5-6.5 points)	7.44 (0.93)
Group 3 (6.5-8.5 points)	8.04 (0.51)
Group 4 (8.5-10 points)	8.27 (1.32)

## Discussion

### Background

Histology is one of the first morphological disciplines faced by medical students. Since it is necessary to integrate basic knowledge from other fields (eg, anatomy, cytology, biology, biochemistry) with spatial awareness, histology is perceived as a difficult subject by most learners. Moreover, students consider histology as irrelevant on their board examination (ie, “Spanish Specialized Health Training examination”) and even for their future clinical practice [21,22]. Most current medical students use social networks daily and demand considerable effort from educators to make the subjects more attractive and dynamic. Creating a social media account is a free educational option that enables access to information and allows users to easily connect with others [23]. Thus, in this work, we analyzed the impact of using a specific Instagram account (@histologiauma) as a teaching resource during a histology course (2022-2023).

### Principal Findings and Implications

Overall, our data demonstrate that medical students who followed and interacted with @histologiauma improved their exam scores compared with those who did not. In fact, a complete lack or a low level of participation generated significant differences in comparison with students who actively engaged with the activity. Most importantly, the enhancement of final grades compared with previous cohorts was not a direct consequence of the extra points awarded to the participants. Thus, improved test performance may serve as indirect and tangible evidence of better long-term knowledge acquisition [24]. These results are supported by the previous opinion of the majority of our students about the positive impact of this experience on academic results. In the first instance, the pre-experiment survey already showed that most of our students believed that social media is rarely used in educational contexts and considered that it may be relevant to include social media platforms as teaching tools, not only to increase accessibility to the content but also to improve their marks. In fact, the results demonstrated a positive disposition toward this innovative approach, since 99.5% of the participants believed they could improve their academic grades thanks to this experience even before participating in it.

Research on the strength or quality of motivation as a predictor of academic success has yielded both definitive and inconclusive findings. In this work, higher engagement and interaction with the content through the proposed interactive activities may have helped in the learning process, which was later reflected in the scores. Indeed, motivation is a determining factor not only for medical students but also for all students to develop sophisticated and successful learning strategies. A study on small group learning found that increased knowledge and understanding of subject matter increase students’ motivation for studying and interest in the course content [24]. The “social constructivist theory” states that socialization can also help students during their personal learning processes [3]. In this sense, social media facilitates active interaction and collaboration by enabling instant communication and motivation [24,25].

Furthermore, our Instagram activities also served as additional virtual tests. Testing is no longer considered as only a tool for evaluation but also for learning [26,27]. Thus, using Instagram for educational purposes incorporates not only these phenomena in the process (as it could have been done through a virtual platform like Moodle) but also other factors that are particularly relevant for current young students: direct interaction with their classmates and immediacy, in addition to their own behavior and daily routine with smartphones and social media. We believe that all these factors increased the motivation and engagement of students with histology, leading to greater retention of the content that was finally reflected as higher scores.

Unfortunately, the information available on social media platforms might not be updated or subjected to peer review; thus, it may be invalid, incorrect, or even false. Conversely, @histologiauma is a platform controlled by our group of specialized teachers, prepared to guide learners toward appropriate knowledge according to the content of the subject. Therefore, the creation of a platform adapted by the teaching staff to the curricular content is ideal not only to boost interest but also to prevent students from accessing unreliable information [28].

Additionally, it is essential to comprehend the preferences of learners in order to create a quality digital learning environment [29,30]. During the experience, the image-based questions, multiple-choice questions, and histological descriptions were considered very useful by the students. Ultimately, this knowledge may help teachers to understand the strengths and weaknesses of the subject matter as well as its impact on adherence.

### Comparison With the Literature

Numerous social media accounts disseminate information about many different types of pathologies to the general public. Although our work focuses on a course within the medical degree program, it is evident that Instagram serves as an optimal and cost-effective platform for capturing attention through passive learning in the field of histology and pathology [31]. In this sense, Nguyen et al [14] reported that 92.5% of students visit Instagram for educational purposes. Accordingly, 97.9% of our respondents strongly believed that social networks should be implemented in higher education.

As far as we know, many accounts share educational content about pathology [16,32], but very few are specifically targeted at histology and assessing the impact of sharing this information on social media on medical students. Another novelty of our approach is that we used Instagram as an educational tool specifically tailored to our students, offering personalized content directly aligned with the course curriculum. Although many other studies have examined the use of social media in education, few have focused on how a targeted, image-based platform like Instagram can enhance engagement and learning outcomes in medical education, particularly in a subject highly reliant on visual materials.

For instance, Essig et al [19] from the School of Medicine at the University of North Carolina created an experience with the Instagram profile @InstaHisto in 2020, which is the most similar to our work in the existing literature. However, one of the main differences between these profiles could be summarized by the word “personalization.” Our private account was created solely and exclusively for second-year students in the medicine degree program at the University of Malaga, in contrast with their public profile. Moreover, they examined the impact of the posts based on the number of views, not focusing on student interaction but rather on the general public. Instead, our work aimed to potentiate students’ knowledge acquisition and to increase engagement with the subject. We also intended to understand students' perceptions about this educational tool. Similarly, the work by Essig et al [19] focused on the National Board of Medical Examiners final exams, reporting that 77% of their students found the histology content from @InstaHisto useful for passing the test. In this line, our survey data reflected a high degree of satisfaction with the utility in the educational environment of these virtual activities (96.5% and 99.3% with multiple-choice questions and image-based questions). More recently, Prabhu and Munawar [11] evaluated 49 Instagram profiles dedicated to the dissemination and teaching of radiology, concluding that it is indeed a better application of this image-based social media platform due to its easy accessibility and appeal to students. In this line, our data reflect that 95% of students believe that using Instagram would enhance their perception of the course and its appeal.

### Limitations

In this work, rating grades increased after use of @histologiauma, even before adding the points awarded for students’ participation in Instagram. It is noticeable that scarce involvement led to no or low improvement, and although there was no significant difference between the most active groups (G2 to G4), we did find a trend toward higher ratings according to the level of participation. In this sense, we cannot completely rule out the influence of other factors masking the impact of this experience, including other curricular or extracurricular activities performed during the school year or personal preferences regarding social media. Nevertheless, as far as we are concerned, standard students from the same academic course share identical academic schedules. All other activities performed during the histology course (such as problem-based learning or the section “From Histology to Medicine”) were developed during theoretical classes or practice and were mandatory. However, we are aware that every academic group is different and repeating the experience during additional academic years would yield more reliable data. Relevant to this, the algorithm used by social media platforms like Instagram tends to favor posts from accounts with which users interact more frequently or with related content, creating information bias for customers [33]. Given this scenario, it is reasonable to interpret that students who interacted with @histologiauma above a threshold were later shown our account or similar profiles in their private feed more often than those who barely engaged or did not participate in the voluntary activity. This interaction likely results in students passively reviewing content each time they visit this platform, which finally positively impacts their acquisition and assimilation of knowledge and therefore their final results. However, this may also minimize the differences between the most active groups.

Another specific limitation of this work is that participating in these activities was not mandatory, which may have led to potential selection bias. We cannot rule out that students following the @histologiauma account were more eager to participate in additional activities than the general medical students. However, the high participation rate (85.6% of students enrolled) notably reduced the impact of this possibility. Even so, eliminating the optional nature of this activity would have yielded clearer data. In addition, it is possible that some of our students do not have an Instagram account because they do not find social networks attractive. Nevertheless, this was rarely the case, since we detected very few exceptions of students who performed the final exam without participating in Instagram (10 of 153 students). For future editions, we intend to propose @histologiauma as an educational instrument in a public mode, encouraging users to create their own hashtags and check the transcendence of the posts.

On the other hand, the use of social media platforms during the educational process of histology should be recommended only as a complement for regular teaching. Evidence that social media is not a panacea was provided in a separate analysis of 131 students who were using the microblog X during class with the aim of fostering student-faculty interaction on two campuses. Although it facilitated discussion, 71% of students found it distracting [34]. For this reason, it is important to find a balance between the usual lecture-based methodology and the inclusion of social media in higher education, not only to meet the curricular needs of students but also to ensure their engagement with their studies.

For future research, the sample size may be broadened to increase the validity and reliability of our findings and include cohorts from other courses or health science degrees such as podiatry, physiotherapy, or nursing. Moreover, specific tests about the content shown on the Instagram account could be implemented. The inclusion of a longitudinal study to track students’ performance and engagement over multiple semesters would allow better understanding of the long-term impact of Instagram-based learning. Although, in general, we detected typical mistakes of pattern recognition of histological structures, a range of accuracy-based rewards could be incorporated into the activity to avoid participation without true commitment. Finally, future experiences could explore the impact of different types of Instagram content (eg, video, live question-and-answer sessions, different quizzes).

### Conclusions

Medical students consider there is inadequate use of social networks for teaching purposes, probably due to a lack of updated methodological approaches in the context of university subjects. Compared with the conventional educational system, social media platforms have a considerable impact on both teachers and students as they offer the possibility to easily connect and collaborate. In fact, one of the main objectives of medical education is to capitalize on the engaging nature of social media tools as part of an overall strategy to use a learner-centered approach. In addition, to increase student engagement during the first year of the degree in Medicine, it is desirable to use attractive didactic methods for learning histology. In this regard, the visual nature of histology is particularly appropriate for the introduction of new image-based tools. Thus, the aim of this study was to investigate an innovative online educational approach for histology based on an Instagram account specifically designed for medical students. In this work, we showed that the use of Instagram has great potential to improve not only the knowledge but also the scores of students of human histology. Our results provide evidence that this teaching strategy boosts students’ learning motivation. In the near future, the classical practical lessons based on the physical microscope might not be enough to meet the needs of medical students. Therefore, Instagram may be considered as a relevant tool for current students to achieve their curricular objectives in a more dynamic, friendly, and enjoyable way under the supervision of the faculty.
